# TRIM5 and the Regulation of HIV-1 Infectivity

**DOI:** 10.1155/2012/426840

**Published:** 2012-05-30

**Authors:** Jeremy Luban

**Affiliations:** Department of Microbiology and Molecular Medicine, University of Geneva, 1211 Geneva, Switzerland

## Abstract

The past ten years have seen an explosion of information concerning host restriction factors that inhibit the replication of HIV-1 and other retroviruses. Among these factors is TRIM5, an innate immune signaling molecule that recognizes the capsid lattice as soon as the retrovirion core is released into the cytoplasm of otherwise susceptible target cells. Recognition of the capsid lattice has several consequences that include multimerization of TRIM5 into a complementary lattice, premature uncoating of the virion core, and activation of TRIM5 E3 ubiquitin ligase activity. Unattached, K63-linked ubiquitin chains are generated that activate the TAK1 kinase complex and downstream inflammatory mediators. Polymorphisms in the capsid recognition domain of TRIM5 explain the observed species-specific differences among orthologues and the relatively weak anti-HIV-1 activity of human TRIM5. Better understanding of the complex interaction between TRIM5 and the retrovirus capsid lattice may someday lead to exploitation of this interaction for the development of potent HIV-1 inhibitors.

## 1. Introduction

HIV-1 was identified only two years after the first report of AIDS in 1981 [1]. The HIV-1 genome was cloned and sequenced, ORFs were identified, and functions of the gene products pinpointed. At a time when few antivirals were in clinical use, HIV-1 proteins were isolated, their activities were described, their structures were determined, and inhibitors were identified [2–5]. The first anti-HIV-1 drug, AZT, was approved for patients in 1987, and effective combinations of anti-HIV-1 drugs were in the clinic by the mid-1990s. Thanks to these anti-HIV-1 drugs, the number of AIDS cases plummeted in countries like the United States. HIV-1 infection became an outpatient disease. Yet, despite the impact of basic science on disease in individuals with HIV-1 infection, the AIDS pandemic has not gone away.

## 2. Ongoing Pandemic and the Need for More Basic Research

Failure to control the AIDS pandemic may be attributable to a number of factors, including the need for improvement in drugs and more ready access to those drugs that already exist. Aside from one extraordinary case of a person who underwent bone marrow transplantation with cells from a CCR5-defective donor [6], there has been no documented cure of HIV-1 infection. Aside from a small effect in one vaccination trial [7], there is no evidence for prevention of HIV-1 infection in people by a vaccine. Without prospects for curative drugs or a preventive vaccine, the cost of HIV-1 infection to individuals and to society will remain high. In New York City there are currently ~110,000 people living with HIV-1 and ~1,600 HIV-related deaths annually (NYC Dept of Health). The toll of AIDS is much greater in medically underserved regions of the world, despite improved distribution of anti-HIV-1 drugs in these places. According to the UNAIDS report concluding in 2010 (http://www.unaids.org/en/), 34 million people were living with HIV infection, and in that year alone there were 2.7 million new infections.

## 3. Host Factors and HIV-1 Infectivity

Much remains to be learned about the function of each of the HIV-1 gene products and the optimization of drugs that inhibit their function. In recent years the focus of much HIV-1 molecular biology research has shifted to host factors that regulate HIV-1 infection. Initially these studies involved searches for host factors that physically interact with individual viral proteins. The cellular proteins cyclophilin A and LEDGF, for example, were found to interact with HIV-1 capsid (CA) and HIV-1 integrase (IN), respectively, [8, 9]. Both of these protein-protein interactions have been studied extensively and have offered novel approaches to HIV-1 inhibition and potential new anti-HIV-1 drug candidates [9–12].

Functional screens have also yielded information concerning host factors that regulate infection by HIV-1 and other retroviruses [13–16]. More recently, several groups have reported human genome-wide RNAi screens to identify factors that regulate HIV-1 infectivity [17–21]. Among host factors identified in these screens are host proteins such as TNPO3 that play critical roles in the poorly understood early events of HIV-1 infection that culminate in establishment of the provirus [15, 22–25]. Ultimately, information springing from the study of any one of these host factors has the potential to be exploited towards the development of drugs that disrupt HIV-1 in people.

## 4. Restriction Factors

Over the past 10 years, in addition to the identification of host factors that promote HIV-1 infectivity, several host factors have been discovered that block HIV-1 infection [26]. Comparative analysis of the genes encoding these proteins, which have been called restriction factors, indicates that some of them have evolved in response to challenge with pathogenic retroviruses [27, 28]. Study of these factors has offered a wealth of information concerning requirements for HIV-1 replication, novel ways that HIV-1 might be targeted therapeutically, potential paths to cure HIV-1 infection, and ways in which innate immune detection of HIV-1 might be amplified to improve vaccination protocols.

## 5. Fv1 and Capsid-Specific Restriction

When HIV-1 and other retroviruses undergo membrane fusion with susceptible target cells, the virion core is released into the target cell cytoplasm. The core of the virion consists of a capsid-protein lattice, within which there are two copies of the viral genome, along with the reverse transcriptase and IN proteins. An extraordinary series of experiments spanning several decades demonstrated that the retroviral CA protein lattice is the viral determinant of sensitivity to a murine-specific restriction factor called Fv1 [29, 30]. Curiously, *Fv1* encodes a retroviral Gag polyprotein [29]. The mechanism of Fv1 restriction is still unknown, but these studies established the concept of retrovirus CA-specific restriction and inspired the search for similar factors targeting HIV-1 CA.

## 6. Cyclophilin A and Capsid-Specific Restriction

Cyclophilin A was the first HIV-1 CA-specific host factor that was identified [9, 31]. Though cyclophilin A is not a restriction factor itself, it controls the accessibility of CA to other host factors that inhibit reverse transcription and other processes essential to the early steps of the infection cycle [32]. One apparent effect of these host factors is to influence these early steps via effects on stability of the HIV-1 virion core [15, 32–36]. The identity of these cyclophilin-regulated host factors is unknown. Additional screens have identified CPSF6 as a conditional regulator of HIV-1 infection, that acts in a capsid-specific manner [15, 37]. CPSF6 is a possible candidate for one such cyclophilin A-regulated restriction factor.

Cyclophilin A cDNAs have retrotransposed many times in evolution, in several cases creating new genes that regulate HIV-1 infectivity in a capsid-specific manner. The first of the cyclophilin A-targeted restriction factors to be identified was the TRIM5-cyclophilin A fusion protein found in South American owl monkeys [38]. A similar, though independently derived, TRIM5-cyclophilin A fusion gene that acts as a capsid-specific restriction factor was created in Asian macaques [39–42]. Nup358/RanBP2, a nuclear pore protein that possesses a cyclophilin A domain also plays a role in HIV-1 infectivity [15, 17, 19, 43].

## 7. The Discovery of TRIM5 as an HIV-1 CA-Specific Restriction Factor

Early studies with HIV-1 showed that infection of cells from nonhuman primates is too inefficient to establish spreading infection [44–48]. It was then shown that dominant-acting inhibitors were present in these species, and that the viral capsid was the main determinant for sensitivity [49–51]. In 2004, two groups independently identified TRIM5 orthologues as being responsible for these species-specific, capsid-specific blocks [38, 52]. The owl monkey orthologue (known as TRIM5-Cyp) targets HIV-1 capsid via its carboxy-terminal cyclophilin A domain [38, 53], and the rhesus macaque orthologue (the alpha isoform) targets HIV-1 capsid via its carboxy-terminal PRY-SPRY domain [52]. Human TRIM5alpha potently restricts EIAV and N-tropic MLV, but it only weakly inhibits HIV-1 lab strains. Differences in specificity between human and macaque TRIM5alpha map to a small block of residues in the PRY-SPRY domain [28, 32, 54, 55]. Though standard HIV-1 lab strains are only weakly inhibited by human TRIM5alpha, some primary HIV-1 isolates are much more sensitive [56, 57].

## 8. The Problem of CA Recognition

**Figure 1:**
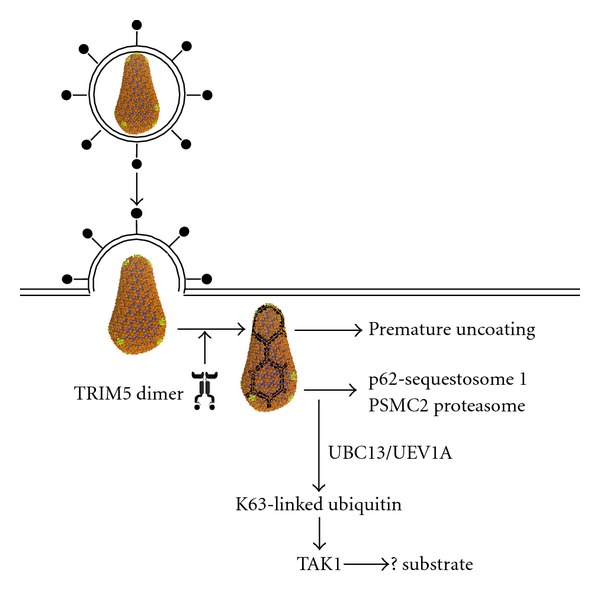
Schematic diagram showing current models of TRIM5-mediated restriction. Free TRIM5 probably exists as a dimer in the target cell cytoplasm. Upon interaction with the capsid of a restriction-sensitive retrovirus, the propensity of TRIM5 to form a complementary hexameric lattice is stimulated. This increases its intrinsic E3 ubiquitin ligase activity. If avidity for the retrovirus capsid is sufficient, the virion core prematurely uncoats and reverse transcription is blocked. Depending upon the proximity of particular cellular E2 enzymes, TRIM5 will either autoubiquitinate and traffic towards proteasomes, or it will activate the TAK1 kinase and downstream signaling molecules.

One of the biggest ongoing challenges for researchers studying TRIM5 is to understand the structural basis for CA recognition. TRIM5 is a multimer, and CA recognition does not occur via a simple protein-protein interaction. Rather, TRIM5 recognizes a complex surface involving the CA lattice [58, 59]. In fact, TRIM5 spontaneously forms a hexameric protein lattice, and this propensity to form a lattice is greatly stimulated in the presence of the CA lattice [60] (Figure 1). This explains why a simple binding assay has not been developed. Extensive efforts have been made by several groups to develop soluble subdomains of the CA lattice that might be used in binding studies [61, 62]. The soluble hexamer unit, for example, seems not to bind to TRIM5 [63, 64]. In contrast, promising results have been obtained with a CA trimer [64]. A requirement for additional host factors such as SUMO-1 may complicate the situation with CA recognition even further [65].

## 9. TRIM5 and E3 Ubiquitin Ligase Activity

At latest count, the human TRIM family comprises ~100 genes [66]. Like other members of this large family, TRIM5 possesses an N-terminal RING domain, a B-box domain, and a coiled-coil domain. The B box and coiled-coil domains promote multimerization of TRIM5 required for restriction activity [67, 68]. The TRIM5 RING domain confers E3 ubiquitin ligase activity, and, in cooperation with certain E2 enzymes, TRIM5 is autocatalytic, covalently attaching ubiquitin to itself [69]. Mutations on the putative E2-interacting face which disrupt this autocatalytic activity block restriction activity [70]. Ubiquitination of TRIM5 contributes to the short half-life of this protein [71], and challenge of cells with viruses bearing restriction-sensitive capsids promotes the proteasome-dependent degradation of TRIM5 [72]. Though TRIM5-stimulated ubiquitination of viral proteins has not been detected, TRIM5 may contribute to the restriction mechanism by recruiting viral components to the proteasome for degradation (Figure 1). TRIM5 interacts biochemically with the proteasome component PSMC2 and colocalizes with proteasomes in infected cells [73]. TRIM5 also associates with the proteasomal adaptor protein p62 [74] though p62 seems to stabilize TRIM5 protein levels.

In certain experimental conditions, restriction activity has been reported in the absence of the RING domain or in the absence of ubiquitination. There are several possible explanations for these discrepancies. One possibility is that, when avidity for a particular CA is great enough, TRIM5 binding to the CA is sufficient to disassemble the virion core prior to reverse transcription [59] (Figure 1). Another possible explanation stems from the fact that TRIM5 blocks multiple steps in the restriction pathway [75]. Disruption of the RING domain rescues the TRIM5-mediated block to reverse transcription and premature uncoating but not subsequent blocks in the infection cycle that lead up to integration [76, 77].

## 10. TRIM5, TAK1, and Inflammation

In combination with the heterodimeric E2, UBC13/UEV1A, TRIM5 catalyzes the synthesis of unattached, K63-linked ubiquitin chains that multimerize and activate the TAK1 kinase complex [63]. These K63-linked ubiquitin chains are not generated by TRIM5 when other E2 enzymes are substituted for UBC13/UEV1A. Disruption of TAK1 or of UBC13/UEV1A prevents restriction activity. Taken together, these observations suggest that the activated TAK1 complex contributes to TRIM5-mediated restriction activity via phosphorylation of a critical cofactor (Figure 1). The identity of this putative cofactor is not known, and direct phosphorylation of CA by TAK1 has not been detected.

Coming at it from another direction, the synthesis of K63-linked ubiquitin chains that activate TAK1 is stimulated by TRIM5 interaction with a restricted capsid lattice [63]. TAK1 activation leads to NF*κ*B and AP-1 signaling which activate inflammatory cytokine transcription. In other words, TRIM5 functions as a pattern recognition receptor specific for the retrovirus capsid lattice. The consequence of TRIM5-mediated signaling for HIV-1-associated inflammation and pathology is only now being considered.

## 11. Future Directions of TRIM5 Research

If a robust assay was developed for TRIM5 interaction with the retrovirus capsid lattice, it would inform attempts to influence HIV-1 CA recognition by TRIM5, and perhaps to develop HIV-1 inhibitors that increase the avidity of this specific interaction. If the avidity of human TRIM5 for the HIV-1 capsid lattice could be increased experimentally, the resulting increase in capsid-stimulated signaling might also be exploited as an adjuvant for anti-HIV-1 immunization.

Recent publicity concerning the apparent cure from HIV-1 infection of a leukemia patient in Berlin with transplantation of cells from a CCR5-mutant donor [6, 78] has generated excitement concerning prospects for curing HIV-1 infection. This case has also renewed interest in basic research concerning gene therapy against HIV-1 and the regulation of HIV-1 latency in people who are already infected with HIV-1. Concerning gene therapy, the most promising approaches at this point involve either disruption of CCR5 [79] or transduction of hematopoietic stem cells with potent HIV-1 restriction factors such as engineered, human TRIM5-cyclophilin A fusion proteins [80].
